# Race/ethnicity and socio-economic differences in colorectal cancer surgery outcomes: analysis of the nationwide inpatient sample

**DOI:** 10.1186/s12885-016-2738-7

**Published:** 2016-09-05

**Authors:** Tomi Akinyemiju, Qingrui Meng, Neomi Vin-Raviv

**Affiliations:** 1 Department of Epidemiology, University of Alabama at Birmingham, 1720 2nd Ave S, Birmingham, AL 35294-0022 USA; 2 Comprehensive Cancer Center, University of Alabama at Birmingham, Birmingham, Alabama USA; 3 University of Northern Colorado Cancer Rehabilitation Institute, Greeley, Colorado USA; 4 School of Social Work, College of Health and Human Sciences, Colorado State University, Fort Collins, Colorado USA

## Abstract

**Background:**

The purpose of this study was to examine racial and socio-economic differences in the receipt of laparoscopic or open surgery among patients with colorectal cancer, and to determine if racial and socio-economic differences exist in post-surgical complications, in-hospital mortality and hospital length of stay among patients who received surgery.

**Methods:**

We conducted a cross-sectional analysis of hospitalized patients with a primary diagnosis of colorectal cancer between 2007 and 2011 using data from Nationwide Inpatient Sample. ICD-9 codes were used to capture primary diagnosis, surgical procedures, and health outcomes during hospitalization. We used logistic regression analysis to determine racial and socio-economic predictors of surgery type, post-surgical complications and mortality, and linear regression analysis to assess hospital length of stay.

**Results:**

A total of 122,631 patients were admitted with a primary diagnosis of malignant colorectal cancer between 2007 and 2011. Of these, 17,327 (14.13 %) had laparoscopic surgery, 70,328 (57.35 %) received open surgery, while 34976 (28.52 %) did not receive any surgery. Black (36 %) and Hispanic (34 %) patients were more likely to receive no surgery compared with Whites (27 %) patients. However, among patients that received any surgery, there were no racial differences in which surgery was received (laparoscopic versus open, *p =* 0.2122), although socio-economic differences remained, with patients from lower residential income areas significantly less likely to receive laparoscopic surgery compared with patients from higher residential income areas (OR: 0.74, 95 % CI: 0.70-0.78). Among patients who received any surgery, Black patients (OR = 1.07, 95 % CI: 1.01-1.13), and patients with Medicare (OR = 1.16, 95 % CI: 1.11-1.22) and Medicaid (OR = 1.15, 95 % CI: 1.07-1.25) insurance experienced significantly higher post-surgical complications, in-hospital mortality (Black OR = 1.18, 95 % CI: 1.00-1.39), and longer hospital stay (Black β = 1.33, 95 % CI: 1.16-1.50) compared with White patients or patients with private insurance.

**Conclusion:**

Racial and socio-economic differences were observed in the receipt of surgery and surgical outcomes among hospitalized patients with malignant colorectal cancer in the US.

## Background

Race/ethnic disparities in healthcare and outcomes among the US colorectal cancer population is well documented, with Blacks experiencing higher incidence and mortality compared with other race/ethnic groups [1–3]. Furthermore, since 1960, colorectal cancer mortality has declined by 39 % among whites, but increased by 28 % among blacks [2]. The increased mortality in blacks with colorectal cancer can be attributed to differences in socioeconomic status (SES) [4–6], tumor biology and stage at diagnosis [7–9], comorbidities [4] treatment [5, 6, 10], post-treatment surveillance [11, 12], physician characteristics [13, 14], and hospital factors [15]. However, despite adjustment for these factors in many studies, Black-White differences in colorectal cancer survival have persisted, worsened and are not fully understood [16–18].

Another predictor of the Black-White differences in survival that has received less attention is the access to and/or utilization of high-quality colorectal cancer treatments. The gap between whites and blacks in colon cancer surgery and chemotherapy has lessened over the years, however, racial differences are still apparent [6, 10]. Compared to whites, black patients were less likely to undergo surgery for colorectal cancer [19–23] and chemotherapy [19–26], and although advances in adjuvant therapy have improved survival in stage III and IV disease [27], surgical resection remains the standard of care for treating and staging non-metastatic colon cancer. A major innovation in surgical techniques was the development of laparoscopic colectomy for colon cancer, which is considered a superior alternative to conventional open colectomy based on findings from randomized trials and meta-analyses [28–31]. These studies have consistently concluded that laparoscopic colectomy is safe, feasible, and associated with many short-term benefits compared with open colectomy. In addition, laparoscopic surgery has been associated with reduction of postoperative pain, length of stay, and early mobilization compared with an open colectomy [29, 32–35].

However while disparities in surgical treatment of colorectal cancer between blacks and whites has been well documented, it is unclear whether those disparities extend to application of new surgical technologies. Several studies that have examined data from the large Nationwide Inpatient Sample (NIS) database have shown inconsistent results regarding the impact of race on colorectal surgical treatment; some studies indicated that Whites were more likely to receive laparoscopic surgery [36], while other studies found that race was not a predictor [30–32]. Many of these previous studies have been using earlier NIS databases (1998–2004), which may be affected by the accuracy of coding for laparoscopic procedures. Furthermore, it remains unclear if the Black-White differences in surgical outcomes (including mortality, post-surgical complications and hospital length of stay) persist after accounting for the type of surgery received.

The aim of this analysis is to examine differences in receipt of colorectal cancer surgery (open and laparoscopic) and hospitalization outcomes among black and white patients hospitalized with a primary diagnosis of colorectal cancer. By utilizing data from the large NIS database and focusing on inpatients that theoretically have successfully accessed the healthcare system, we simultaneously control for differences in access to care as well as other potential confounders including demographic factors, tumor characteristics, and comorbidities. Determining the influence of race/ethnicity on the type of surgical colorectal cancer treatment received, and associated cancer outcomes may help to further shed light on the persistent disparities in colorectal cancer outcomes between black and white patients in the U.S, highlighting areas where targeted efforts may be focused to improve survival for all colorectal cancer patients.

## Methods

This is a cross-sectional analysis of hospitalized patients between 2007 and 2011 with a primary diagnosis of colorectal cancer. The inpatient data were obtained from the Health Cost and Utilization Project Nationwide Inpatient Sample (HCUP-NIS). The HCUP-NIS is a large all-payer inpatient care database covering over 1000 hospitals in the U.S., with data on over seven million hospitals stays per year [37]. The HCUP-NIS database contains clinical and nonclinical data elements for each hospital stay, including clinical variables for all diagnoses and procedures occurring during admission. Non-clinical variables are also included, such as median household income in the patient’s zip code, rural/urban residence, and expected payment source. More information on HCUP-NIS can be obtained at: https://www.hcup-us.ahrq.gov/nisoverview.jsp.

### Clinical variables

Primary diagnosis of malignant colorectal cancer was captured using International Classification of Disease, Ninth edition (ICD-9) codes (153.X, 154.0-154.3, 154.8). We created a proxy colorectal cancer stage variable, classifying malignant colorectal cancer patients into metastatic and non-metastatic (ICD-9 codes: 196.X, 197.X, 198.X) since the HCUP dataset does not include cancer stage variables. For the major comorbid conditions, we created a modified Deyo comorbidity index using ICD-9 codes. The conditions included cerebrovascular disease, congestive heart failure, chronic pulmonary disease, diabetes mellitus with or without chronic complications, dementia, myocardial infarctions, peripheral vascular disease, rheumatic disease, peptic ulcer disease, mild liver disease, hemiplegia or paraplegia, renal disease, moderate or severe liver disease, and HIV/AIDS. The presence of each condition within each patient was identified. A single comorbidity score was created as the sum of the number of conditions per patient, and this approach of using the Charleston index as modified by Deyo has been previously examined in the NIS database [38–40].

### Individual variables

Other covariates used in the analysis include race/ethnicity, categorized into White, Black, Hispanic, and Other (Other included Asians, Pacific Islanders, Native Americans and Other races combined due to low sample sizes), residential income, insurance type and residential region. Residential income was divided into quartiles ranging from the lowest income to the highest income based on median household income at the zip-code level. Residential region was categorized into large metropolitan areas (metropolitan areas with 1 million residents or more), small metropolitan areas (metropolitan areas with less than 1 million residents), micropolitan areas (Non-metropolitan areas adjacent to metropolitan areas) and non-metropolitan or micropolitan areas (noncore areas with or without its own town) using the 2003 version of the Urban Influence Codes [41]. Insurance status was classified into Medicaid, Medicare, private (includes Blue Cross, commercial carriers, private HMOs and PPOs, and self-insured) and others (includes Worker’s Compensation, Title V, and other government programs) [37].

### Outcome measures

There were two main objectives of this study. First was to examine racial and socio-economic differences in the receipt of laparoscopic or open surgery procedures among patients with malignant colorectal cancer; and second, to determine racial and socio-economic differences in post-surgical complications, in-hospital mortality and hospital length of stay among patients who received colorectal laparoscopic or open surgery. Our analyses were based on two datasets, the full dataset with all colorectal cancer patients, and the reduced dataset with only patients who received laparoscopic or open surgery. ICD-9 procedure codes were used to identify laparoscopic (ICD-9 codes: 17.33-17.36, 17.39, 45.81, 48.42, 48.51) and open (ICD-9 codes: 45.7X, 45.80, 45.82, 48.43, 48.52, 48.62, 48.63) surgery. The length of hospital stay was calculated by subtracting the admission date from the discharge date with same-day stays coded as 0. In-hospital mortality was identified as deaths occurring during hospitalization. ICD-9 diagnosis codes were used to identify the presence of post-surgical complications, which include mechanical wounds, infections, urinary, pulmonary, gastrointestinal, cardiovascular and intra-operative complications. Since the dataset only includes information collected during hospital admissions, our analysis excluded complications and mortality occurring after hospital discharge.

### Statistical analysis

We examined the race/ethnicity and socio-economic differences in study characteristics using Chi-square tests for categorical variables and ANOVA for continuous variables (age, length of stay, number of comorbidities). Multinomial logistic regression analysis was conducted to determine the association between laparoscopic surgery and open surgery versus no surgery and logistic regression analysis was conducted to determine the association between laparoscopic surgery versus open surgery among those who received any surgery, and adjusted for race/ethnicity, age, sex, diagnosis year, stage, residential income, insurance type, and residential region.). To examine the associations between race/ethnicity and residential income with post-operative complications, logistic regression was restricted to patients who received surgery adjusting for race/ethnicity, age, sex, diagnosis year, stage, residential income, insurance type, and residential region. Linear regression models were computed to examine the associations with hospital length of stay using the reduced dataset. All statistical analyses were conducted in SAS 9.4.

## Results

A total of 122,631 hospitalized patients were identified with a primary diagnosis of malignant colorectal cancer between 2007 and 2011. Among them, 17,327 (14.13 %) had laparoscopic surgery, 70,328 (57.35 %) received open surgery, while 34976 (28.52 %) did not receive any surgery. Table 1 shows the socio-demographic and clinical distributions of study participants by race. The majority of patients were White (74 %), while (11.8 %) were Black, 7.3 % were Hispanic and 6.4 % were of Other race. White patients were older at the time of admission (mean age: 68.8) compared with Blacks (mean age 63.8), Hispanics (mean age 63.5) and Other racial groups (mean age 65.4), and the majority of Black patients (50.4 %) lived in the lowest residential income areas compared with 22.0 % of White, 36.1 % of Hispanic and 19.7 % of Other races. There were also racial differences in the clinical variables. White patients were less likely to present with metastatic disease (34.8 %) compared with Blacks (40.8 %), Hispanics (35.5 %) and other racial groups (36.8 %). White patients were also more likely to receive laparoscopic or open surgery compared with other racial groups; 26.5 % of Whites received no surgery compared with 36.4 % of Blacks, 33.9 % of Hispanics and 31.3 % of Other racial groups. However, White patients were more likely to have two or more post-surgical complications (8.5 %) compared with 7.9 % of Blacks, 6.7 % of Hispanics and 6.1 % of Other racial groups.

**Table 1:** Distribution of baseline characteristics by race among colorectal cancer patients, Nationwide Inpatient Sample, 2007-2011

	Race			
Study Characteristics N (%)/ Mean (SD)	White (*N* = 91344)	Black (*N* = 14500)	Hispanic (*N* = 8930)	Other (*N* = 7857)
Sex				
Female	45203 (49.5)	7542 (52.0)	4074 (45.6)	3816 (48.6)
Male	46136 (50.5)	6958 (48.0)	4856 (54.4)	4039 (51.4)
Age at admission (years)	68.8 (13.8)	63.8 (13.6)	63.5 (14.6)	65.4 (14.3)
Length of Stay (days)	8.2 (7.2)	9.4 (9.2)	8.5 (7.8)	8.2 (8.1)
Number of Comorbidities	0.4 (0.7)	0.4 (0.7)	0.3 (0.7)	0.3 (0.6)
Residential income				
First Quartile-Lowest	19691 (22.0)	7090 (50.4)	3121 (36.1)	1482 (19.7)
Second Quartile	19691 (22.0)	3026 (21.5)	1975 (22.8)	1637 (21.8)
Third Quartile	22656 (25.3)	2264 (16.1)	2148 (24.8)	1919 (25.5)
Fourth Quartile-Highest	23834 (26.6)	1680 (12.0)	1413 (16.3)	2484 (33.0)
Insurance Type				
Medicare	54489 (59.7)	6779 (46.8)	3865 (43.3)	3511 (44.7)
Medicaid	3663 (4.0)	1917 (13.2)	1430 (16.0)	1028 (13.1)
Private	28720 (31.4)	4351 (30.0)	2593 (29.0)	2658 (33.8)
Other	4472 (4.9)	1453 (10.0)	1042 (11.7)	660 (8.4)
Residential Region				
Large Metro	43411 (54.1)	9507 (70.3)	6514 (77.1)	5537 (75.4)
Small Metro	25867 (32.2)	2927 (21.6)	1521 (18.0)	1320 (18.0)
Micropolitan	11027 (13.7)	1099 (8.1)	418 (5.0)	490 (6.7)
Stage at presentation				
Non-Metastatic	59539 (65.2)	8586 (59.2)	5757 (64.5)	4967 (63.2)
Metastatic	31805 (34.8)	5914 (40.8)	3173 (35.5)	2890 (36.8)
Surgery				
Laparoscopic	13285 (14.5)	1766 (12.2)	1231 (13.8)	1045 (13.3)
Open	53844 (59.0)	7459 (51.4)	4669 (52.3)	4356 (55.4)
No surgery	24215 (26.5)	5275 (36.4)	3030 (33.9)	2456 (31.3)
Complications				
0	61525 (67.4)	10096 (69.6)	6630 (74.2)	5918 (75.3)
1	21678 (23.7)	3254 (22.4)	1700 (19.0)	1459 (18.6)
> =2	8141 (8.9)	1150 (7.9)	600 (6.7)	480 (6.1)
Died during Hospitalization				
No	87932 (96.3)	13843 (95.5)	8623 (96.7)	7563 (96.3)
Yes	3342 (3.7)	649 (4.5)	299 (3.4)	291 (3.7)

Table 2 presents the results of multivariable logistic regression models examining factors associated with the receipt of laparoscopic or open surgery against no surgery, adjusted for age, sex, diagnosis year, race, income, stage, insurance, residential region and comorbidities. There were significant differences in receipt of surgery by age, sex, race/ethnicity, income, stage, insurance, region and comorbidities (*p <* .0001). Compared with males, females were significantly (*p <* .0001) more likely to receive both laparoscopic (OR = 1.19, 95 % CI: 1.14-1.24) and open surgery (OR = 1.10, 95 % CI: 1.07-1.13), and Black (laparoscopic OR = 0.74, 95 % CI: 0.69-0.79; open OR = 0.75, 95 % CI: 0.72-0.79), Hispanic (laparoscopic OR = 0.88, 95 % CI: 0.82-0.95; open OR = 0.83, 95 % CI: 0.79-0.88) and Other racial group (laparoscopic OR: 0.85, 95 % CI: 0.79-0.93; open OR = 0.90, 95 % CI: 0.86-0.96) patients were significantly less likely to receive surgery compared with White patients. In addition, compared with patients residing in the highest residential income areas, those in lower residential income areas were significantly less likely to receive laparoscopic (OR = 0.64, 95 % CI: 0.60-0.68) and open (OR = 0.86, 95 % CI: 0.82-0.90) surgery. However, among patients that received any surgery, there were no significant racial differences in which surgery was received (laparoscopic versus open, *p =* 0.2122), although socio-economic differences remained, with patients from lower residential income areas significantly less likely to receive laparoscopic surgery compared with patients from higher residential income areas (OR: 0.74, 95 % CI: 070–0.78).

**Table 2:** Multivariable logistic regression models of Laparoscopic Surgery and Open Surgery, Nationwide Inpatient Sample, 2007-2011

	Laparoscopic^a^		Open Surgery^a^			Laparoscopic vs. Open Surgery^b^	
	*N*	OR (95 % CI)	*N*	OR (95 % CI)	*P-*value	OR (95 % CI)	*P-*value
Age	19221	0.99(0.93, 0.99)	84429	0.99 (0.99, 0.98)	<.0001	1.00 (0.99, 1.00)	0.7739
Sex					<.0001		<.0001
Male	9280	Ref	42324	Ref		Ref	
Female	9878	1.19 (1.14, 1.24)	41983	1.10 (1.07, 1.13)		1.09 (1.05, 1.13)	
Race/Ethnicity					<.0001		0.2122
White	13285	Ref	53054	Ref		Ref	
Black	1766	0.74 (0.69, 0.79)	7325	0.75 (0.72, 0.79)		0.97 (0.91, 1.04)	
Hispanic	1231	0.88 (0.82, 0.95)	4606	0.83 (0.79, 0.88)		1.05 (0.98, 1.13)	
Other	1045	0.85 (0.79, 0.93)	4311	0.90 (0.86, 0.96)		0.95 (0.88, 1.03)	
Residential Income					<.0001		<.0001
Q4-Highest	5285	Ref	19219	Ref		Ref	
Q3	4985	0.86 (0.82, 0.91)	20253	0.97 (0.93, 1.01)		0.89 (0.85, 0.94)	
Q2	4619	0.78 (0.74, 0.83)	21873	0.90 (0.86, 0.94)		0.86 (0.82, 0.91)	
Q1-Lowest	3953	0.64 (0.60, 0.68)	21347	0.86 (0.82, 0.90)		0.74 (0.70, 0.78)	
Stage					<.0001		<.0001
Non-Metastatic	14865	Ref	56421	Ref		Ref	
Metastatic	4356	0.37 (0.35, 0.38)	29246	0.66 (0.64, 0.68)		0.57 (0.54, 0.59)	
Insurance Type					<.0001		<.0001
Private	6884	Ref	27472	Ref		Ref	
Medicare	10863	0.94 (0.89, 0.99)	48239	0.99 (0.96, 1.04)		0.94 (0.89, 0.99)	
Medicaid	693	0.28 (0.25, 0.31)	4283	0.48 (0.46, 0.51)		0.57 (0.51, 0.63)	
Other	781	0.34 (0.31, 0.38)	4435	0.56 (0.53, 0.59)		0.63 (0.57, 0.69)	
Residential Region					<.0001		<.0001
Large metro	10950	Ref	41199	Ref		Ref	
Small metro	4974	0.99 (0.94, 1.04)	23398	1.18 (1.14, 1.22)		0.83 (0.79, 0.87)	
Micropolitan	1699	0.82 (0.76, 0.88)	10561	1.25 (1.20, 1.31)		0.64 (0.60, 0.69)	
Comorbidities					<.0001		<.0001
0	13721	Ref	58363	Ref		Ref	
1	4115	0.79 (0.75, 0.83)	19119	0.89 (0.86, 0.92)		0.89 (0.84, 0.93)	
≥ 2	1385	0.64 (0.59, 0.69)	6947	0.80 (0.76, 0.85)		0.81 (0.75, 0.87)	

Adjusted for age, sex, diagnosis year, race, income, stage, insurance, residential region and comorbidities

^a^Multinomial regression model for laparoscopic surgery and open surgery versus no surgery

^b^Multivariable logistic regression model comparing laparoscopic surgery versus open surgery among CRC patients who received surgery

Table 3 presents the results of multivariable analysis of post-surgical outcomes among colorectal cancer patients who received either laparoscopic or open surgery. There were significant differences in the odds of post-surgical complications by race (*p =* 0.0021), socio-economic (*p* = 0.0472) and insure type (*p* < .0001). Post-surgical complications were significantly higher among Black patients (OR = 1.07, 95 % CI: 1.01-1.13), but lower among Hispanic patients (OR = 0.93, 95 % CI: 0.87-0.99) compared with White patients. Patients with Medicare (OR = 1.16, 95 % CI: 1.11-1.22) and Medicaid (OR = 1.15, 95 % CI: 1.07-1.25) insurance types also experienced more post-surgical complications compared with those with private insurance. There were also racial differences in mortality outcomes, with Black patients more likely to experience in-hospital mortality (OR = 1.18, 95 % CI: 1.00-1.39) compared with Whites. In addition, patients residing in the lowest residential income areas (OR: 1.30, 95 % CI: 1.11-1.51) and patients without private insurance (OR: 1.95, 95 % CI: 1.49-2.56) were more likely to experience in-hospital mortality.

**Table 3:** Multivariable logistic regression analysis of outcomes after colorectal cancer surgery, Nationwide Inpatient Sample, 2007-2011

Study Characteristics	N (%)	Post-Surgical Complications^a^ OR (95 % CI)	*P-*value	In-Hospital Mortality^a^ OR (95 % CI)	*P-*value
Age	104834	1.01 (1.01, 1.01)	<.0001	1.05 (1.05, 1.06)	<.0001
Sex			<.0001		<.0001
Male	52193 (49.8)	Ref		Ref	
Female	52510 (50.2)	0.77 (0.73, 0.81)		0.72 (0.65, 0.79)	
Race/Ethnicity			0.0021		0.0364
White	66339 (76.6)	Ref		Ref	
Black	9091 (10.5)	1.07 (1.01, 1.13)		1.18 (1.00, 1.39)	
Hispanic	5837 (6.7)	0.93 (0.87, 0.99)		1.01 (0.82, 1.24)	
Other	5356 (6.2)	0.96 (0.90, 1.02)		0.79 (0.63, 1.01)	
Residential Income			0.0472		0.0080
Q4-Highest	24504 (24.1)	Ref		Ref	
Q3	25238 (24.9)	1.05 (1.01, 1.10)		1.19 (1.03, 1.36)	
Q2	26492 (26.1)	1.01 (0.96, 1.06)		1.20 (1.04, 1.39)	
Q1-Lowest	25300 (24.9)	0.99 (0.95, 1.04)		1.30 (1.11, 1.51)	
Stage			<.0001		<.0001
Non-Metastatic	5397 (13.5)	Ref		Ref	
Metastatic	24142 (60.3)	1.17 (1.13, 1. 21)		1.80 (1.63, 1.99)	
Insurance Type			<.0001		<.0001
Private	34356 (33.6)	Ref		Ref	
Medicare	59102 (57.0)	1.16 (1.11, 1.22)		1.23 (1.05, 1.45)	
Medicaid	4976 (4.8)	1.15 (1.07, 1.25)		1.66 (1. 23, 2.22)	
Other	5216 (5.0)	1.03 (0.95, 1.12)		1.95 (1.49, 2.56)	
Residential Region			0.0116		0.6859
Large metro	52149 (56.2)	Ref		Ref	
Small metro	28372 (30.6)	0.95 (0.91, 0.99)		0.96 (0.86, 1.08)	
Micropolitan	12260 (13.2)	1.01 (0.95, 1.06)		0.95 (0.80, 1.08)	
Comorbidities			<.0001		<.0001
0	72084 (69.6)	Ref		Ref	
1	23234 (22.4)	1.19 (1.14, 1.23)		1.86 (1.67, 2.08)	
≥ 2	8332 (8.0)	1.19 (1.13, 1.26)		3.11 (2.73, 3.54)	

^a^Adjusted for age, sex, diagnosis year, race, income, stage, insurance, residential region and comorbidities

Furthermore, Black patients (β = 1.33, 95 % CI: 1.16-1.50) experienced significantly longer hospital stay compared with Whites, as did patients of lower residential income areas (β = 0.84, 95 % CI: 0.68-1.00). Patients with Medicaid (β = 2.91, 95 % CI: 2.66-3.16) and other insurance types (β = 1.72, 95 % CI: 1.47-1.96) had approximately up to 3.5 days longer hospital stays, respectively, compared with patients with private insurance. Conversely, patients in small metropolitan (β = −0.36, 95 % CI: −0.48 to −0.24) and micropolitan areas (β = −0.71, 95 % CI; −0.88 to −0.54) had significantly shorter hospital stays compared with patients in large metropolitan areas (Table 4).

**Table 4:** Multivariable linear regression analysis of in-hospital length of stay after colorectal cancer surgery, Nationwide Inpatient Sample, 2007-2011

	In-Hospital Length of stay^ab^ (95 % CI)	*P-*value
Age	0.06 (0.05, 0.06)	<.0001
Sex		<.0001
Male	Ref	
Female	−0.55 (−0.66,-0.45)	
Race/Ethnicity		<.0001
White	Ref	
Black	1.33 (1.16, 1.50)	
Hispanic	0.18 (−0.02, 0.39)	
Other	0.09 (−0.12, 0.30)	
Residential Income		<.0001
Q4-Highest	Ref	
Q3	0.33 (0.18, 0.47)	
Q2	0.37 (0.22, 0.52)	
Q1-Lowest	0.84 (0.68, 1.00)	
Stage		<.0001
Non-Metastatic	Ref	
Metastatic	1.76 (1.65, 1.87)	
Insurance Type		<.0001
Private	Ref	
Medicare	0.66 (0.51, 0.81)	
Medicaid	2.91 (2.66, 3.16)	
Other	1.72 (1.47, 1.96)	
Residential Region		<.0001
Large metro	Ref	
Small metro	−0.36 (−0.48,-0.24)	
Micropolitan	−0.71 (−0.88,-0.54)	
Comorbidities		<.0001
0	Ref	
1	1.52 (1.40, 1.65)	
≥ 2	3.01 (2.82, 3.21)	

^a^Length of hospital stay was calculated by subtracting the admission date from the discharge date with same-day stays coded as 0

^b^Adjusted for age, sex, diagnosis year, race, income, stage, insurance, residential region and comorbidities

## Discussion

In this study we examined race/ethnicity and SES disparities in colorectal cancer surgery and post-surgical outcomes among hospitalized patients in the large Nationwide Inpatient Sample dataset, representative of hospitalized patients in the U.S. Our analysis of hospitalized patients, who have successfully accessed healthcare revealed that there remained significant racial and SES disparities in the receipt and type of colorectal cancer surgery as well as subsequent clinical outcomes. Black patients were less likely to receive any type of surgery compared with other racial groups, however, among patients that received surgery, there were no racial differences but significant socio-economic differences in the type of surgery received. Patients of lower residential income areas, those with Medicaid or Other insurance types, and patients residing outside of large metropolitan areas were less likely to receive laparoscopic surgery. These differences may account for the racial and socio-economic differences observed in post-surgical complications, in-hospital mortality and hospital length of stay.

Starting in the late 1980s and throughout the 1990s, reports appeared in the literature describing the inequalities in dissemination of new treatments for colorectal cancer and other cancer experienced by minority populations, especially Blacks, in the United States [19–26], fostering interest as to why these racial discrepancies exist. Multiple factors are believed to contribute to differences in surgical treatment among colorectal cancer patients, including disease characteristics, comorbidities, patients’ demographic factors, factors related to the health system, and surgeon experience [42–44]. Similar to other studies within the NIS patient population databases [30–32, 36], our findings suggest that non-White patients remained less likely to receive any surgery compared with White patients, although among those who did receive surgery, there were no racial differences in the type of surgery received. One possible explanation is that laparoscopic surgery is often performed on younger patients with less complicated disease, possibly reflecting the individual surgeon’s comfort level with the procedure [45]. We observed an independent influence of socio-economic status on type of surgery received, suggesting that patients with higher socio-economic status are the most likely recipients laparoscopic surgery. It remains an open question whether these patients also happen to be the most ideal candidate for this surgery type based on their disease status and other comorbidities; we did not observe an independent association between number of comorbidities and type of surgery received after adjusting for race and residential income.

Black patients and patients of lower socio-economic status experienced worse hospitalization outcomes, with more post-surgical complications, in-hospital mortality and longer hospital stay compared with Whites and patients of other race. Furthermore, worse outcomes were observed among residents of lower residential income areas, and patients with non-private insurance. These findings provide additional evidence of the disproportionate burden of colorectal cancer morbidity and mortality among Black and low-SES populations [46–51], which is not necessarily explained by differential access to healthcare since hospitalized patients have theoretically already accessed the health system. Our findings also corroborate studies in the literature suggesting that having health insurance does not uniformly increase access or use of health care services [52, 53]. We observed an independent influence of insurance type on outcomes even after adjusting for race and residential income; patients without private insurance, usually obtained through employment, were less likely to receive surgery, and those that did receive any surgery were less likely to receive laparoscopic surgery. Patients on Medicare and Medicaid may experience difficulties in finding healthcare providers, since reimbursement rates for these insurance types are usually significantly lower than those offered by private insurance [3, 54]. Thus, patients with non-private insurance may present at advanced disease stages, experience delayed treatment, may be offered less expensive treatment options, and/or may have other health-related conditions making them less suitable candidates for surgery [55–57]. Other factors such as cultural beliefs, patient preferences and social support may also exert significant influences on treatment choice, type, and outcome. More subjective factors such as quality of patient-physician communication, discrimination, and capacity to navigate health system bureaucracies may also play a role in treatment outcome, even among hospitalized patients already within the healthcare system [58].

Although our study benefited from large sample sizes and objective measures of diagnoses and procedures, there are some limitations associated with this observational study using administrative data. The NIS database is discharge specific and does not allow long-term follow-up at the patient level. ICD-9-CM diagnostic and procedure codes were used to identify procedures examined in the study, and the possibility of coding errors and missing procedure or diagnosis codes exists. Furthermore, we could not discern whether some of the racial differences in treatment were due to personal patient preferences, thus future studies are needed to fully explore the extent to which patient preference influences type of treatment and outcomes. We were unable to assess non-surgical forms of treatment such as chemotherapy and radiotherapy, as detailed information regarding these data items are not readily available in HCUP. Finally, in order to be effective at capturing socioeconomic gradients in cancer outcomes, several studies used a measure of census tract or census block with a priori cut-points [59–61]. However, due to patient privacy concerns, residential level SES was only provided at the zip-code level, therefore this could likely lead to an underestimation of our SES estimates.

## Conclusion

There were racial and socio-economic differences observed in the receipt of surgery, and surgical outcomes among hospitalized patients with malignant colorectal cancer. Although laparoscopic surgery for colorectal cancer is now widely accepted as the treatment of choice for colorectal cancer, further studies are needed to better understand factors associated with treatment type that may be racially patterned, including individual and physician level factors that may influence the treatment decisions. In addition, future studies are needed to identify reasons underlying differences in the receipt of laparoscopic surgery by insurance coverage and residential region. Determining whether these differences are due to limited availability of trained personnel and/or surgical equipment, high out-of-pocket costs, or other reasons may help inform policies designed to eliminate such barriers, ultimately improving hospitalization outcomes for all patients with colorectal cancer.
